# Analgesic Prescribing Trends of Opioids, Nonsteroidal Anti-Inflammatory Drugs, and Paracetamol in Taiwan (2001‐2020): Age-Period-Cohort Cross-Sectional Study

**DOI:** 10.2196/92534

**Published:** 2026-09-01

**Authors:** Chun-Ji Lin, Chia-Shuan Chang, Chih-Yi Wu, Jiun-Yi Wang, I-Ching Lin, Hung-Yi Chiou

**Affiliations:** 1 Department of Healthcare Administration, College of Medical and Health Science, Asia University, 500, Lioufeng Rd., Wufeng, Taichung, 41354, Taiwan; 2 Department of Behavioral and Community Health, School of Public Health, University of Maryland, College Park, Maryland, MD, United States; 3 Institute of Population Health Sciences, National Health Research Institutes, 35, Keyan Road, Zhunan, Miaoli, 350401, Taiwan, 886 037-206-166 ext 36301; 4 Department of Family Medicine, Asia University Hospital, Taichung, Taiwan; 5 Department of Kinesiology, Health, and Leisure, Chienkuo Technology University, Changhua, Taiwan; 6 School of Public Health, College of Public Health, Taipei Medical University, Taipei, Taiwan

**Keywords:** analgesics, population health surveillance, pharmacovigilance, age-period-cohort analysis, real-world data, opioid stewardship, age differences, adolescent analgesics

## Abstract

**Background:**

Despite ongoing global changes in analgesic prescribing practices, long-term trends accounting for age, period, and cohort effects remain poorly understood in Asian populations, where opioid and nonopioid prescribing trajectories differ from those observed in Western nations.

**Objective:**

This study aimed to examine the long-term prescribing trends of opioids, nonsteroidal anti-inflammatory drugs (NSAIDs), and paracetamol in Taiwan by age, calendar period, and birth cohort using an age-period-cohort (APC) framework.

**Methods:**

We conducted a population-based repeated cross-sectional study using Taiwan National Health Insurance claims data (2001‐2020). The cohort included all insured individuals aged 1 to 85 years, with an annual population size of 22.3 to 23.1 million. Exposure variables were structured for APC modeling, comprising 5-year age groups, four 5-year calendar periods (2001‐2020), and 5-year birth cohorts (1920‐1924 to 2015‐2019).

**Results:**

Opioid prescribing prevalence escalated from 123.66 per 100,000 persons in 2001 to 2005 to 706.93 in 2016 to 2020. APC analysis showed a significant positive net drift for opioids (+10.93% per year, 95% CI 10.36%-11.51%; *P*<.001). The estimated magnitude of the opioid prescribing increase was sensitive to the prescription frequency threshold used. In contrast, paracetamol and NSAIDs showed overall declines (net drifts: −2.44%, 95% CI −2.58% to −2.29%, and −0.82%, 95% CI −0.97% to −0.66%, respectively). Notably, local drifts revealed a significant increase in NSAID prescribing only among adolescents aged 11 to 15 years (+0.80% per year, 95% CI 0.34%-1.27%). Generational analysis suggested higher opioid prescribing risk among younger cohorts (rate ratios 3.06 for the 2015‐2019 cohort), although the increase relative to the 2000 to 2004 reference cohort was not statistically significant.

**Conclusions:**

Reimbursed analgesic prescribing in Taiwan has diverged across drug classes, with a consistent increase in opioid prescribing across all ages and a shift toward NSAID prescribing in younger populations. These claims-based trends should be interpreted as reimbursed prescribing rather than total analgesic use. These findings may inform targeted pharmacovigilance and policies to ensure appropriate pain management while mitigating potential opioid-related risks.

## Introduction

Analgesic prescribing is a core component of pain management and a main objective for pharmacovigilance and population health surveillance. Prescribing patterns are shaped by multiple interacting forces, including demographic aging, evolving clinical guidelines, reimbursement policies, health care access, and medication safety concerns [1,2]. Consequently, long-term monitoring of reimbursed analgesic prescribing at the population level is essential for identifying changes in prescribing practice and potential safety priorities [2-5].

Over the past two decades, rising opioid-related harms in North America and Europe have prompted broad stewardship initiatives, including guideline revisions, prescribing restrictions, and enhanced monitoring systems, with several settings reporting subsequent stabilization or declines in opioid prescribing [2,6-8]. However, trends have not been uniform across regions. In fact, opioid use in parts of Asia has continued to increase rather than decline [9-12], highlighting the need for context-specific surveillance. Nonopioid analgesics also have distinct trade-offs between clinical efficacy and safety profiles. For example, while some studies found that nonsteroidal anti-inflammatory drugs (NSAIDs) may offer more potent and sustained analgesia than paracetamol (acetaminophen) [13,14], NSAID use is also associated with risks of gastrointestinal bleeding, renal injury, and cardiovascular events [15,16]. For paracetamol, although it is often favored for its relative safety, it still carries a risk of dose-related hepatotoxicity [17]. Analgesic needs and clinical approaches also vary significantly across the life course, ranging from pediatric populations to older adults with multimorbidity [18,19], and are increasingly influenced by generational shifts in attitudes toward pain management. These multifaceted influences highlight the need for a comprehensive assessment of both opioid and nonopioid prescribing to fully capture the evolving landscape of analgesic exposure across different demographic and generational subgroups.

Despite growing attention to analgesic prescribing trends, recent studies have shown that opioid and nonopioid prescribing patterns vary across health systems and population groups [1,20]. However, many studies still focus on a single analgesic class or report only aggregate secular trends, potentially obscuring important heterogeneity across age groups and birth cohorts [7,10,20-22]. Analyses that simultaneously account for age, calendar period, and birth cohort, such as age-period-cohort (APC) approaches, are also needed to disentangle these interrelated temporal dimensions and identify specific age groups or cohorts with disproportionate changes in prescribing risk [23-25]. The limited number of studies jointly considering these factors may inflate observed associations by conflating biological aging with period-specific and generational effects [26]. To address these gaps, we used nationwide Taiwan National Health Insurance (NHI) claims data from 2001 to 2020 to characterize and compare long-term prescribing patterns of opioids, NSAIDs, and paracetamol. Using an APC model, we estimated age, period, and cohort effects to determine whether prescribing trajectories diverged across analgesic classes and identify specific age groups or birth cohorts that may warrant intensified monitoring and targeted stewardship.

## Methods

This study followed the STROBE (Strengthening the Reporting of Observational Studies in Epidemiology) reporting guidelines for cross-sectional studies. The completed STROBE checklist is provided in Checklist 1.

### Study Design and Data Source

A population-based repeated cross-sectional design was used for the current study, and our data were drawn from 2 national databases in Taiwan. For medical use data, we used the Taiwan National Health Insurance Research Database (NHIRD), which is managed by the National Health Insurance Administration. The NHIRD covers more than 99% of the population and contains comprehensive records of outpatient services, hospitalizations, and prescriptions, providing a robust source for prescribing surveillance. For population data, we drew from the Department of Household Registration, Ministry of the Interior.

### Ethical Considerations

This study was approved by the Research Ethics Committee of the National Health Research Institutes (EC1111106-E-R2). Because this study used secondary claims data and aggregated population data, the requirement for informed consent was waived by the ethics committee. The NHIRD contains deidentified claims records, and all analyses were conducted using anonymized or deidentified data to protect participant privacy and confidentiality. No participants were contacted, recruited, or compensated in this study.

### Study Population and Observation Period

The analytic sample consisted of NHIRD medical claims data from January 1, 2001, to December 31, 2020, providing a continuous 20-year observation period for examining long-term trends in reimbursed analgesic prescribing. Our study included all insured individuals who were aged between 1 and 85 years. The population at risk was estimated using the year-end population counts from the Ministry of the Interior. Depending on the year, the annual study population ranged from 22,266,932 to 23,078,937 from 2001 to 2020. The NHI policy approved equitable access to health care and limits financial incentives related to the choice of prescription drugs. Our study excluded those with missing or unknown gender and birth date data.

### Measures

Our study defined prescribed analgesic drugs as medications with recognized pain-relieving effects based on prior studies, clinical practice, and evidence-based pain management guidelines [20]. Specifically, pain-related medications are commonly categorized as opioids, paracetamol, NSAIDs, and gabapentinoids [20]. Although gabapentinoids are officially classified as antiepileptic agents, they are known to produce analgesic effects in clinical settings; however, their use in Taiwan is limited due to restrictions under the NHI system [27]. Therefore, our study excluded gabapentinoids and focused solely on paracetamol, NSAIDs, and opioids.

Analgesic prescriptions were identified using the Anatomical Therapeutic Chemical classification system. In our analysis, opioids corresponded to code N02A, NSAIDs corresponded to code M01A, and paracetamol corresponded to codes N02BE01 and N02BE51. Repeated analgesic prescribing was defined as receiving at least 3 prescriptions from the same analgesic class within a calendar year [28,29].

We grouped age, period, and cohort into 5-year intervals, resulting in 17 age groups (1‐5, 6‐10, ..., and 81‐85 years), 4 period groups (2001‐2005, 2006‐2010, 2011‐2015, and 2016‐2020), and 20 birth cohort groups (1920‐1924, ..., and 2015‐2019). For each age group within each period, we estimated the prevalence per 100,000 population. In addition, we calculated age-standardized prevalence using the population of 2001 to 2005 as the standard population. To obtain age-standardized prevalence per 100,000 persons, the age-specific prevalence estimates for each period were weighted accordingly.

### Statistical Analysis

To evaluate temporal trends and age-, period-, and cohort-specific patterns in repeated reimbursed analgesic prescribing, we organized the 2001 to 2020 observation period into four 5-year calendar periods for APC analysis. We then used a 2-step analytical approach. We first conducted a model selection process based on the analysis of deviance as proposed by Clayton and Schifflers [23,24]. We fitted a series of nested APC specifications and used deviance-based tests to assess the incremental contribution of age, period, and cohort effects. The model fit statistics are summarized in Table S1 in Multimedia Appendix 1. Next, given the perfect linear dependency among age, period, and cohort (age = period – cohort), which prevents unique estimation in standard linear regression models, we used the estimable function approach described by Rosenberg et al [25] to address this identification problem. This approach provides interpretable APC summary measures that can be estimated despite the linear dependency among age, period, and cohort rather than relying on uniquely estimated absolute age, period, and cohort effects [25]. Using this approach, we summarized temporal dynamics in prescribing prevalence through 4 APC measures: net drift, local drifts, period rate ratios (RRs), and cohort RRs. Net drift was interpreted as the overall annual percentage change in age-adjusted prescribing prevalence over calendar time, summarizing the combined linear period and cohort trend within the APC framework. Local drifts represented age-specific annual percentage changes, indicating whether prescribing trends increased or decreased differently across age groups. Period RRs represented relative prescribing prevalence in each calendar period compared with the 2001 to 2005 reference period adjusted for age and nonlinear cohort effects. Cohort RRs represented relative prescribing prevalence in each birth cohort compared with the reference birth cohort adjusted for age and nonlinear period effects. Additional age-, period-, and cohort-specific estimates supporting these APC measures are provided in Table S2 in Multimedia Appendix 1. In this study, the 2001 to 2005 period category and the age group of 1 to 5 years were used as the reference categories. Because birth cohort is determined by calendar period and age in the APC framework, this reference age-period combination corresponded to the 2000 to 2004 birth cohort, which was therefore used as the reference cohort. Statistical significance was evaluated using the Wald test with 2-tailed hypotheses and an α value of .05. To assess the influence of the prescription frequency threshold on the APC estimates, we conducted a sensitivity analysis using a broader definition of at least one prescription from the same analgesic class within a calendar year. The results of this sensitivity analysis can be found in Table S3 in Multimedia Appendix 1. All statistical analyses were conducted using SAS (version 9.4; SAS Institute) and R (version 4.3.2; R Foundation for Statistical Computing).

## Results

### Characteristics and APC Model

Age-specific 5-year period prevalence rates of analgesic prescribing are shown in Table 1. Throughout the study period, NSAIDs and paracetamol were the predominant choice for analgesic prescribing. In the initial period (2001‐2005), their prevalence rates were substantially higher than those of opioids, ranging from 13,052.95 to 51,952.64 for NSAIDs and from 20,397.55 to 54,805.04 for paracetamol per 100,000 persons across age groups. However, the prescribing patterns shifted distinctively over time across drug classes. Both NSAIDs and paracetamol analgesics showed downward patterns by 2016 to 2020, with NSAIDs dropping to between 12,287.48 and 42,051.40 per 100,000 persons and paracetamol dropping to between 13,722.56 and 47,693.16 per 100,000 persons, respectively. Opioid use escalated substantially across all age groups, with prevalence rates rising from a range of 0.36 to 807.97 per 100,000 persons in 2001 to 2005 to a range of 1.00 to 9942.95 per 100,000 persons in 2016 to 2020.

**Table 1.** Age-specific 5-year period prevalence of repeated nonsteroidal anti-inflammatory drug (NSAID), paracetamol, and opioid prescribing among National Health Insurance beneficiaries aged 1 to 85 years in Taiwan (2001‐2020).

Age group (year)	Prescription prevalence rate per 100,000 persons			
	2001‐2005	2006‐2010	2011‐2015	2016‐2020
NSAIDs				
1‐5	51,952.64	48,169.08	52,680.01	41,617.47
6‐10	24,217.17	25,313.47	28,616.67	21,563.29
11‐15	14,105.66	14,628.97	17,203.90	15,678.69
16‐20	13,466.47	12,239.74	13,680.08	13,212.11
21‐25	13,052.95	12,334.92	12,702.94	12,287.48
26‐30	15,251.62	13,755.37	15,065.67	14,043.83
31‐35	18,252.57	15,821.96	17,168.10	17,368.36
36‐40	19,258.78	16,890.76	17,537.40	17,831.89
41‐45	19,496.24	17,582.59	17,601.88	16,523.89
46‐50	20,949.83	18,885.51	19,137.86	17,145.84
51‐55	22,782.96	20,738.72	20,862.06	19,165.11
56‐60	26,713.84	22,128.42	22,261.86	20,369.48
61‐65	30,744.43	26,843.39	24,131.75	22,147.01
66‐70	34,350.20	31,092.55	29,237.85	23,902.19
71‐75	35,476.30	33,335.88	31,911.23	27,271.13
76‐80	33,138.54	32,397.95	31,769.27	28,033.47
81‐85	43,177.27	40,757.05	43,307.29	42,051.40
Paracetamol				
1‐5	54,805.04	47,749.79	46,800.10	34,870.73
6‐10	34,179.53	31,261.83	30,072.42	19,717.46
11‐15	25,800.41	23,503.86	22,988.54	16,757.48
16‐20	23,311.37	19,836.47	19,373.07	14,977.87
21‐25	20,397.55	18,681.90	17,579.56	13,722.56
26‐30	21,846.40	18,980.07	19,557.73	14,957.33
31‐35	23,925.21	19,640.98	20,075.26	17,268.34
36‐40	23,619.40	19,459.89	18,634.64	16,017.20
41‐45	22,870.09	19,525.99	17,995.14	14,123.04
46‐50	23,780.99	20,698.83	19,462.23	14,737.30
51‐55	25,230.82	22,732.19	21,588.89	16,954.16
56‐60	29,356.79	24,276.50	23,590.93	18,885.78
61‐65	32,449.95	28,310.23	25,007.26	20,949.41
66‐70	35,431.97	31,779.13	29,462.70	22,458.44
71‐75	35,883.83	33,938.91	32,094.42	26,283.92
76‐80	33,277.46	33,015.64	32,940.62	28,016.85
81‐85	44,304.00	43,262.79	48,449.17	47,693.16
Opioids				
1‐5	0.363551	0.79863	0.925144	1.002668
6‐10	0.592314	1.280099	2.003292	2.496567
11‐15	2.590302	4.846901	8.33832	8.340548
16‐20	7.941273	22.24344	35.80282	40.9002
21‐25	16.31995	45.84773	73.96435	78.85145
26‐30	33.65626	77.96135	127.8943	132.7993
31‐35	57.15419	126.1066	205.9964	235.5716
36‐40	86.86481	200.6227	314.3916	386.2125
41‐45	121.001	287.2094	463.0504	558.2152
46‐50	163.247	395.6203	649.4524	803.9249
51‐55	215.5913	536.5244	870.8949	1085.473
56‐60	289.2118	718.9867	1127.085	1375.968
61‐65	375.1389	984.8498	1520.900	1801.301
66‐70	479.9140	1359.107	2300.359	2444.945
71‐75	559.7899	1742.142	3196.521	3638.863
76‐80	586.9455	2023.311	3939.797	4767.857
81‐85	807.9685	3143.605	7037.366	9942.947

A similar trend was observed for age-standardized prevalence. Specifically, the rates for NSAIDs showed a slight overall decline from 22,222.19 to 19,200.22 per 100,000 persons, and paracetamol also followed a steady downward trajectory from 27,624.88 to 18,271.32 per 100,000 persons. Conversely, opioids surged dramatically, increasing from 123.66 to 706.93 per 100,000 persons over the study period.

On the basis of goodness-of-fit statistics (Table S1 in Multimedia Appendix 1), the full APC model yielded the lowest deviance values among all 3 drug classes (NSAIDs: 50,744.95; paracetamol: 50,184.21; opioids: 1041.15), indicating a significantly superior fit compared with any reduced specifications. The analysis of deviance revealed that removing any single temporal dimension led to a substantial increase in deviance (*P*<.001 in all cases), suggesting that the reduced models failed to adequately explain the data. This indicates that the observed analgesic prescribing patterns could not be sufficiently characterized by age, period, or cohort alone or by any 2D reduced specification. Therefore, the full APC model was retained to characterize long-term prescribing trends across all 3 temporal dimensions.

### Overall Trends (Net Drifts) and Period Effects

**Figure 1.**
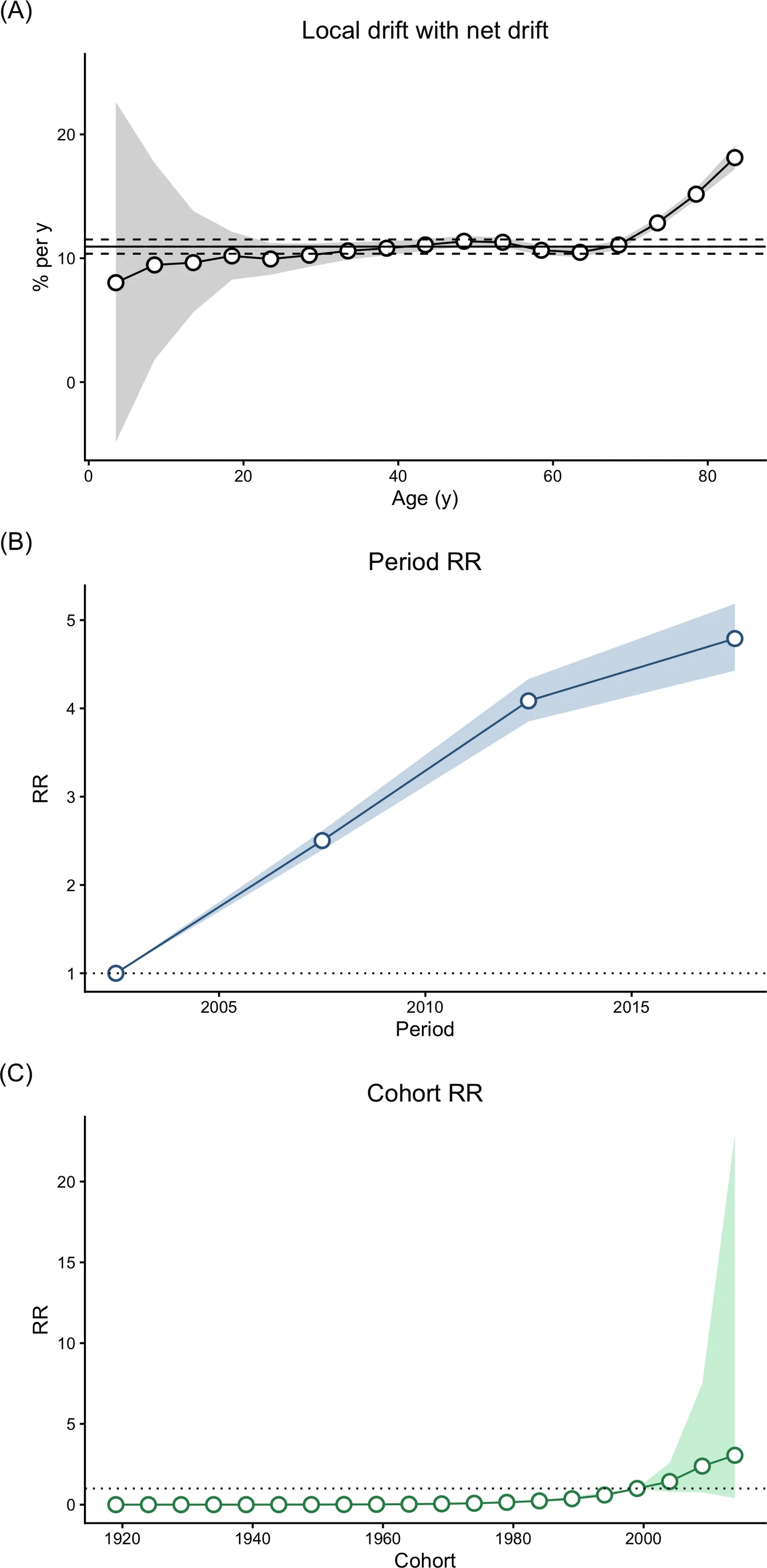
Age-, period-, and cohort-specific patterns in repeated opioid prescribing among National Health Insurance beneficiaries aged 1 to 85 years in Taiwan (2001‐2020). Panel (A) shows age-specific local drifts, expressed as annual percentage changes in opioid prescribing. Panel (B) shows period rate ratios (RRs) relative to the 2001 to 2005 reference period. Panel (C) shows cohort RRs relative to the 2000 to 2004 reference birth cohort. Opioid local drift estimates are displayed separately from those of nonsteroidal anti-inflammatory drugs and paracetamol because their substantially larger magnitude would compress the nonopioid curves and reduce visual readability if plotted on the same scale.

Net drifts are shown in Table 2, and period RRs are shown in Table 3 and Figure 1B. Overall, the annual percentage changes varied significantly by drug class. NSAIDs and paracetamol exhibited decreasing trends, with net drifts estimated at −0.82% per year (95% CI −0.97% to −0.66%) for NSAIDs and −2.44% per year (95% CI −2.58% to −2.29%) for paracetamol. Conversely, opioids showed a substantial increasing trend, with the net drift estimated at 10.93% per year (95% CI 10.36%-11.51%). Over the study period, period RRs for NSAIDs relative to the 2001 to 2005 baseline declined, reaching 0.91 (95% CI 0.88-0.93) in 2006 to 2010, 0.94 (95% CI 0.92-0.96) in 2011 to 2015, and 0.86 (95% CI 0.84-0.88) in 2016 to 2020. Similarly, period RRs for paracetamol compared to baseline showed a progressive decline, falling to 0.87 (95% CI 0.85-0.89) in 2006 to 2010, 0.84 (95% CI 0.82-0.86) in 2011 to 2015, and 0.67 (95% CI 0.65-0.68) in 2016 to 2020. Conversely, period RRs for opioids increased substantially in each subsequent period, rising to 2.50 (95% CI 2.39-2.61) in 2006 to 2010, 4.08 (95% CI 3.85-4.33) in 2011 to 2015, and 4.79 (95% CI 4.42-5.18) in 2016 to 2020. Sensitivity analysis using a broader definition of one or more prescriptions showed that the positive opioid net drift remained evident, although attenuated (+6.76% per year, 95% CI 6.49%-7.03%). This finding supports the directional robustness of the increasing opioid prescribing trend but indicates that the estimated magnitude was threshold sensitive (Table S3 in Multimedia Appendix 1).

**Table 2.** Estimated net drifts and age-specific local drifts in repeated nonsteroidal anti-inflammatory drug (NSAID), paracetamol, and opioid prescribing among National Health Insurance beneficiaries aged 1 to 85 years in Taiwan (2001‐2020).

	NSAIDs, % change per year (95% CI)	Paracetamol, % change per year (95% CI)	Opioids, % change per year (95% CI)
Net drift	−0.82 (−0.97 to −0.66)	−2.44 (−2.58 to –2.29)	10.93 (10.36 to 11.51)
Age-specific trends (year)			
1‐5	−1.34 (−1.75 to −0.92)	−2.94 (−3.37 to −2.52)	8.02 (−4.82 to 22.61)
6‐10	0.04 (−0.36 to 0.46)	−2.76 (−3.14 to −2.38)	9.45 (1.80 to 17.69)
11‐15	0.80 (0.34 to 1.27)	−2.41 (−2.80 to −2.02)	9.64 (5.62 to 13.80)
16‐20	0.39 (−0.07 to 0.86)	−2.55 (−2.94 to −2.17)	10.18 (8.27 to 12.13)
21‐25	−0.14 (−0.60 to 0.30)	−2.37 (−2.75 to −1.99)	9.93 (8.66 to 11.21)
26‐30	−0.18 (−0.61 to 0.24)	−2.02 (−2.39 to −1.64)	10.24 (9.31 to 11.18)
31‐35	−0.06 (−0.46 to 0.32)	−1.91 (−2.27 to −1.55)	10.59 (9.88 to 11.30)
36‐40	−0.61 (−0.99 to −0.24)	−2.60 (−2.95 to −2.24)	10.81 (10.24 to 11.37)
41‐45	−1.03 (−1.40 to −0.65)	−3.02 (−3.38 to −2.66)	11.07 (10.59 to 11.55)
46‐50	−1.12 (−1.49 to −0.74)	−2.91 (−3.27 to −2.55)	11.36 (10.94 to 11.79)
51‐55	−1.04 (−1.41 to −0.66)	−2.46 (−2.83 to −2.10)	11.28 (10.89 to 11.68)
56‐60	−1.64 (−2.04 to −1.23)	−2.64 (−3.03 to −2.25)	10.64 (10.25 to 11.02)
61‐65	−2.17 (−2.58 to −1.75)	−2.83 (−3.23 to −2.42)	10.47 (10.10 to 10.85)
66‐70	−2.33 (−2.78 to −1.88)	−2.87 (−3.31 to −2.43)	11.06 (10.70 to 11.43)
71‐75	−1.59 (−2.09 to −1.09)	−1.85 (−2.34 to −1.36)	12.85 (12.46 to 13.23)
76‐80	−0.81 (−1.41 to −0.22)	−0.72 (−1.30 to −0.13)	15.17 (14.72 to 15.62)
81‐85	−0.18 (−1.04 to 0.68)	0.26 (−0.57 to 1.11)	18.12 (17.20 to 19.05)

**Table 3.** Estimated period rate ratios (RRs) for repeated nonsteroidal anti-inflammatory drug (NSAID), paracetamol, and opioid prescribing among National Health Insurance beneficiaries aged 1 to 85 years in Taiwan (2001‐2020). Period RRs are estimated relative to the 2001 to 2005 reference period.

	NSAIDs, RR (95% CI)	Paracetamol, RR (95% CI)	Opioids, RR (95% CI)
2001-2005 (reference)	1.00	1.00	1.00
2006-2010	0.91 (0.88-0.93)	0.87 (0.85-0.89)	2.50 (2.39-2.61)
2011-2015	0.94 (0.92-0.96)	0.84 (0.82-0.86)	4.08 (3.85-4.33)
2016-2020	0.86 (0.84-0.88)	0.67 (0.65-0.68)	4.79 (4.42-5.18)

### Age-Specific Divergence (Local Drifts)

**Figure 2.**
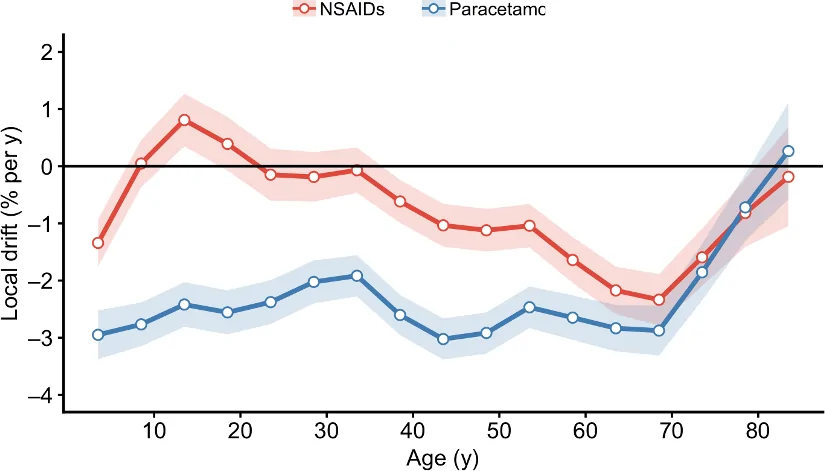
Age-specific annual percentage changes in repeated nonsteroidal anti-inflammatory drug (NSAID) and paracetamol prescribing among National Health Insurance beneficiaries aged 1 to 85 years in Taiwan (2001‐2020). The curves represent the estimated local drift (annual percentage change) across age groups for NSAIDs (red) and paracetamol (blue) derived from age-period-cohort models. Shaded ribbons indicate 95% CIs. The solid horizontal black line at 0% marks the threshold of no change; values above this line indicate an increasing trend, whereas values below indicate a decreasing trend.

Different age-specific local drifts were identified for each analgesic class (Figure 1, Figure 2 and Table 2). Among NSAIDs, trends varied across age groups, with local drifts ranging from −2.33% (95% CI −2.78% to −1.88%) to +0.80% (95% CI 0.34%-1.27%) per year. Children and adolescents generally exhibited positive local drifts across age groups. Specifically, estimates were 0.04% (95% CI −0.36% to 0.46%) for the ages of 6 to 10 years, 0.80% (95% CI 0.34%-1.27%) for the ages of 11 to 15 years, and 0.39% (95% CI −0.07% to 0.86%) for the ages of 16 to 20 years. Notably, only the age group of 11 to 15 years showed a statistically significant increase. In contrast, adult and older populations showed declining local drifts (minimum of −2.33%, 95% CI −2.78% to −1.88%), whereas the oldest age group (81‐85 years) showed a positive but nonsignificant trend.

Paracetamol use showed an overall decline across the life course, with age-specific variation. Specifically, local drifts ranged from −2.94% (95% CI −3.37% to −2.52%) to −2.76% (95% CI −3.14% to −2.38%) among children, from −2.55% (95% CI −2.94% to −2.17%) to −2.41% (95% CI −2.80% to −2.02%) among adolescents, and from −3.02% (95% CI −3.38% to −2.66%) to −1.91% (95% CI −2.27% to −1.55%) among adults aged 31 to 60 years. The magnitude of decline was most pronounced in the age group of 41 to 45 years. Similar to the patterns observed for NSAIDs, the age group of 81 to 85 years exhibited the only positive yet nonsignificant trend.

Opioid use showed an overall increasing trend across the age spectrum, with statistically significant effects in nearly all age groups. The only exception was children aged 1 to 5 years, where the increase was not statistically significant. The magnitude of these annual percentage changes generally trended upward with age, reaching 18.12% (95% CI 17.20%-19.05%) in the oldest age group (81‐85 years).

### Generational Shifts

**Figure 3.**
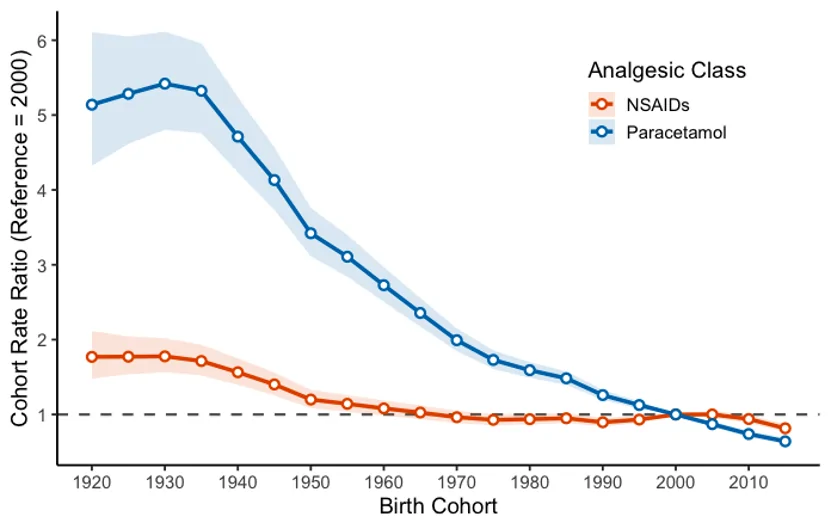
Estimated cohort rate ratios (RRs) for repeated nonsteroidal anti-inflammatory drug (NSAID) and paracetamol prescribing among National Health Insurance beneficiaries aged 1 to 85 years in Taiwan (2001‐2020). The birth cohort of 2000 to 2004 was selected as the reference group (RR=1.0). The graph presents cohort RRs for repeated reimbursed prescribing in each birth cohort using the 2000 to 2004 birth cohort as the reference group and accounting for age and period components in the age-period-cohort model.

Generational analyses, depicted in Figures 1 and 3 and Table S2 in Multimedia Appendix 1, highlight distinct shifts in prescribing risks over time. For paracetamol, a significant downward trend characterized the generational profile. The RRs dropped substantially from the earliest cohorts (1920-1924), decreasing from 5.13 (95% CI 4.32‐6.10) to 0.64 (95% CI 0.59‐0.68) for the most recent cohort (2015-2019); notably, the lack of overlap between these CIs underscores the statistical significance of this decline within the model.

Trends for NSAIDs generally mirrored this downward trajectory but with distinct nuances. While the RRs declined from 1.76 (95% CI 1.47‐2.11) in the earliest cohorts to 0.81 (95% CI 0.76‐0.87) for the most recent group, the trend was not entirely monotonic. The 2005 to 2009 NSAID cohort diverged from the decline, presenting a positive risk that, though not statistically significant, marked a notable interruption in the overall downward trend.

Opioids stood apart with a pervasive upward shift. Among cohorts preceding the reference group (born in 2000‐2004), the relative risk remained significantly lower than the reference yet displayed a distinct upward trajectory, rising from 0.0001 (95% CI 0.0001‐0.0001) in the 1920 to 1924 cohort to 0.60 (95% CI 0.46‐0.77) in the 1995 to 1999 cohort. Following the reference cohort, the point estimates were higher among cohorts born after 2005, reaching 3.06 for the 2015 to 2019 cohort; however, these increases were not statistically significant relative to the 2000 to 2004 reference cohort.

## Discussion

### Principal Findings

To the best of our knowledge, this is the first population-based study using APC analysis to examine the temporal dynamics of analgesic prescribing in Taiwan over a two-decade period. We identified divergent prescribing trends across analgesic classes. While nonopioid analgesics generally exhibited downward trajectories, the decline in NSAID prescribing rate (net drift: −0.82% per year) was notably slower than that of paracetamol (net drift: −2.44% per year), with NSAIDs paradoxically showing increasing trends among children and adolescents. Conversely, opioid prescribing demonstrated a substantial rising trend, with a net drift of 10.93% per year. In addition, our generational analysis revealed a significant overall cohort effect in opioid prescribing, with the cohort RR curve showing an upward pattern across successive birth cohorts. Furthermore, a distinct generational shift was observed in the prescribing of nonopioid analgesics. Among younger generations, prescribing patterns have shifted toward an NSAID-predominant approach; specifically, the likelihood of being prescribed paracetamol has diminished substantially, whereas NSAID prescribing has remained relatively stable or increased.

In contrast to international trends [8,9,21,30], our study identified a continuous and dramatic surge in the likelihood of opioid prescribing in Taiwan. While research using APC to examine opioid prescribing trends across the lifespan remains limited, a study in the United States analyzing individuals aged 16 years and older between 1999 and 2018 identified period effects characterized by an initial increase followed by a subsequent decline [8]. Notably, while the US data indicated a decline in opioid use following its peak around 2015 [8], the likelihood of opioid prescribing in our study continued to escalate without abatement.

Our results align with those of previous Taiwan studies that have identified increasing trends. Specifically, research covering 2002 to 2007 reported an initial rise [31], and a subsequent study analyzing the 2008 to 2018 period observed a linear increase of less than 20% [10]. However, our results highlighted the importance of applying APC modeling to control for age and cohort effects as the conventional estimates without APC may significantly obscure the true severity of the surge. After controlling for age and cohort effects, the period effect intensified dramatically, with the likelihood of opioid prescribing increasing nearly 5-fold in the most recent period (period RR 4.79) compared to 2001 to 2005.

While prior research indicates that most opioid prescriptions in Taiwan were administered to patients with cancer [10], data from the Taiwan Cancer Registry (2001‐2020) show that the age-standardized incidence rate of invasive cancer increased by only 21.7% over the study period [32]. This discrepancy suggests that increased prescribing is not solely driven by rising cancer prevalence but likely reflects expanded pain treatment services and evolving standards for noncancer pain management. In addition, although the NHI reimbursement regulations regarding patient eligibility remained largely unchanged between 2001 and 2020, the formulary expanded with the inclusion of 75 new opioid items between 2001 and 2020 [27,33]. That is, the observed increase may not be due to relaxed eligibility criteria but may relate to other factors, such as broader medication options, the expansion of pain management services, high health care accessibility, and a growing emphasis on patient autonomy [10,34,35]. Therefore, continuous surveillance is necessary to ensure reasonable and appropriate opioid prescribing practices [36,37].

Consistent with studies conducted in France and the Netherlands [1,6], our study identified a general decline in nonopioid analgesic prescribing. Notably, this decline differed by drug class, with paracetamol showing a substantially steeper decrease (net drift: −2.44% per year) than NSAIDs (−0.82% per year). Theoretically, these declining trends could be attributed to a reduction in the incidence of diseases requiring analgesia [17,38]. However, this possibility appears unlikely as epidemiological data indicate that the incidence of upper respiratory infections, otitis media, migraine, and tension-type headache remained stable whereas osteoarthritis and low back pain showed an increasing trend in Taiwan throughout the observation period [39]. Another possible explanation lies in the impact of reimbursement policies. According to Article 51 of the National Health Insurance Act, over-the-counter medications are not included in insurance coverage [40]. Following this mandate, the National Health Insurance Administration implemented a policy of gradually phasing out reimbursement for these items since 2005 [41]. Consequently, the decline in nonopioid analgesics likely reflects a shift in acquisition channels.

After controlling for period and cohort effects, our findings indicated that NSAID prescribing showed an increasing trend during adolescence that was statistically significant, specifically in the age group of 11 to 15 years. Specifically, there is mounting evidence suggesting that sleep deficiency is associated with hyperalgesia changes and altered pain processing [42,43]. Given that Taiwanese adolescents have experienced rising rates of sleep problems and inadequate sleep in recent years [44], such chronic sleep deficit among Taiwanese adolescents may facilitate central sensitization, thereby lowering pain thresholds and increasing the demand for analgesia. In addition, obesity and musculoskeletal strain are relatively common among Taiwanese adolescents [45-47], and these conditions may further exacerbate pain sensitivity as obesity-related systemic inflammation can amplify nociceptive responses and increase pain perception [48]. Altogether, the growing use of NSAIDs among younger individuals may reflect their effectiveness in managing inflammation-related pain in the context of increased pain susceptibility.

Furthermore, psychosocial dynamics play a critical role. For adolescents, education demands are high, and falling behind due to absence is a significant concern [49]. Under such pressure, guardians often prioritize antipyretics with superior and prolonged efficacy [50]. This preference aligns with clinical evidence favoring NSAIDs over paracetamol for pediatric pain management [13,14]. Thus, the rising trend in NSAID prescribing may reflect a collective pursuit by adolescents, caregivers, and clinicians for stronger and longer-acting interventions to minimize the disruption of disease on daily routines within a fast-paced, highly competitive society. The proposed mechanisms remain exploratory, and this adolescent-specific NSAID prescribing pattern should be interpreted cautiously given that only the age group of 11 to 15 years reached statistical significance. Nevertheless, this observed rise in NSAID prescribing among adolescents may represent a meaningful deviation warranting continued surveillance.

A central policy implication of applying an APC framework to repeated reimbursed analgesic prescribing is that it helps distinguish broad temporal changes from prescribing patterns concentrated in specific age groups or birth cohorts. In this study, the sustained increase in opioid prescribing, the pronounced increases among older adults, and the adolescent-specific increase in NSAID prescribing indicate where continued surveillance may be particularly relevant within Taiwan’s health system. These findings may therefore provide a more targeted basis for future studies using longitudinal NHIRD data to examine clinically meaningful medication-related outcomes, including adverse drug events, opioid-related hospitalizations, and dependence.

### Limitations

This study has several limitations. NHIRD claims lack clinical detail (eg, pain severity, duration, and indications) and do not support dose-based metrics. Therefore, our outcomes should be interpreted as repeated reimbursed prescribing events rather than treatment intensity or indication-specific analgesic use. Because this study analyzed population-level analgesic prescribing trends, it could not determine the relative contributions of cancer-related and non–cancer-related pain management to opioid prescribing trends. In addition, because the primary definition required at least 3 prescriptions from the same analgesic class within a calendar year, short-term or episodic analgesic prescribing, including acute postsurgical opioid prescriptions, may have been underestimated. APC analyses are subject to identification constraints, and 5-year groupings may smooth short-term changes and yield less stable boundary cohort estimates; accordingly, we emphasize long-term patterns. In addition, our APC models were fitted to aggregated age-by-period data; therefore, results should be interpreted at the population level and do not support individual-level inference. Given the ecological nature of this population-level APC analysis, our study could not directly confirm the underlying causes of the observed analgesic prescribing trends. Although we discussed several potential mechanisms and contextual factors, these explanations may not be exhaustive. Moreover, although younger age groups showed generally positive NSAID local drifts, only the age group of 11 to 15 years reached statistical significance, whereas the adjacent age groups of 6 to 10 years and 16 to 20 years did not. Therefore, population-level interpretations of adolescent-specific NSAID prescribing patterns should be made with particular caution. Finally, analyses were restricted to 3 reimbursed analgesic classes and did not capture over-the-counter or out-of-pocket purchases, so declines in reimbursed nonopioid prescribing may partly reflect channel shifts. As this was a population-based repeated cross-sectional study in a single-payer system, causal inference and generalizability to other health systems are limited.

### Conclusions

In this population-based APC analysis of reimbursed prescribing in Taiwan (2001‐2020), analgesic prescribing trends diverged markedly across drug classes: opioid prescribing increased substantially, whereas reimbursed prescribing of paracetamol and NSAIDs declined overall, with a steeper reduction in paracetamol. However, the magnitude of the estimated opioid prescribing increase was sensitive to the prescription frequency threshold used. Age- and cohort-specific estimates revealed pronounced heterogeneity, including rising NSAID prescribing in adolescents and a sustained increase in opioid prescribing in older adults, with evidence of higher opioid prescribing risk in more recent birth cohorts. These findings support continued surveillance and stewardship of opioid prescribing and highlight the need for future studies incorporating clinical indications, dose intensity, and nonreimbursed acquisition channels to clarify underlying drivers.

## Supplementary material

10.2196/92534 Multimedia Appendix 1 Goodness-of-fit statistics of age-period-cohort models, estimated cohort rate ratios for analgesic utilization, and sensitivity analysis results.

10.2196/92534 Checklist 1 STROBE checklist.

## Abbreviations

APC: age-period-cohort
NHI: National Health Insurance
NHIRD: National Health Insurance Research Database
NSAID: nonsteroidal anti-inflammatory drug
RR: rate ratio
STROBE: Strengthening the Reporting of Observational Studies in Epidemiology
