# Supplementary material for: Analgesic Prescribing Trends of Opioids, Nonsteroidal Anti-Inflammatory Drugs, and Paracetamol in Taiwan (2001‐2020): Age-Period-Cohort Cross-Sectional Study
Source: JMIR Public Health Surveill. 2026 Sep 1;12:e92534. doi: 10.2196/92534 (PMC13533038; doi:10.2196/92534)
Supplement: Supplementary file 2 [file publichealth-v12-e92534-s001.docx]

**Supplementary Table 1.** Goodness-of-fit of age–period–cohort model assessment for NSAIDs, Paracetamol and Opioids prevalence.

| Models | df | Deviance | Likelihood-Ratio Statistic | *p*-Value |
| --- | --- | --- | --- | --- |
| NSAIDs |  |  |  |  |
| Age | 51 | 578103.1799 | 527358.2282(21) | <.0001 |
| Period | 64 | 13606430.7000 | 13555685.7483(34) | <.0001 |
| Cohort | 48 | 4507903.9615 | 4457159.0098(18) | <.0001 |
| Age–Period | 48 | 285954.8621 | 235209.9104(18) | <.0001 |
| Age–Cohort | 32 | 126372.6996 | 75627.7479(02) | <.0001 |
| Period–Cohort | 45 | 4131051.2887 | 4080306.3370(15) | <.0001 |
| Age–Period–Cohort | 30 | 50744.9517 | reference | reference |
| Paracetamol | | | | |
| Age | 51 | 2313032.1804 | 2262847.9725(21) | <.0001 |
| Period | 64 | 8772787.5230 | 8722603.3151(34) | <.0001 |
| Cohort | 48 | 5190934.6409 | 5140750.4330(18) | <.0001 |
| Age–Period | 48 | 211952.4445 | 161768.2366(18) | <.0001 |
| Age–Cohort | 32 | 211221.0541 | 161036.8462(02) | <.0001 |
| Period–Cohort | 45 | 2830961.3427 | 2780777.1348(15) | <.0001 |
| Age–Period–Cohort | 30 | 50184.2079 | reference | reference |
| Opioids | | | | |
| Age | 51 | 683360.6555 | 682319.5067(21) | <.0001 |
| Period | 64 | 4035230.9536 | 4034189.8048(34) | <.0001 |
| Cohort | 48 | 1657379.9709 | 1656338.8221(18) | <.0001 |
| Age–Period | 48 | 19459.9297 | 18418.7809(18) | <.0001 |
| Age–Cohort | 32 | 60261.6567 | 59220.5079(02) | <.0001 |
| Period–Cohort | 45 | 31928.3136 | 30887.1648(15) | <.0001 |
| Age–Period–Cohort | 30 | 1041.1488 | reference | reference |

df: degree of freedom. Likelihood-ratio statistic: increase in deviance from the age–period–cohort model. df *: increase in df from the age–period–cohort model.

**Supplementary Table 2.** Estimated cohort rate ratios (RR) and 95% confidence intervals for analgesic utilization by birth cohort in Taiwan, derived from Age-Period-Cohort models.

|  | NSAIDs  RR (95% CI) | | Paracetamol  RR (95% CI) | | Opioids  RR (95% CI) | |
| --- | --- | --- | --- | --- | --- | --- |
| 1920-1924 | 1.76 | (1.47,2.11) | 5.13 | (4.32,6.10) | 0.0001 | (0.0001,0.0001) |
| 1925-1929 | 1.77 | (1.53,2.04) | 5.28 | (4.61,6.05) | 0.0003 | (0.0002,0.0004) |
| 1930-1934 | 1.77 | (1.56,2.01) | 5.41 | (4.80,6.11) | 0.0006 | (0.0005,0.0009) |
| 1935-1939 | 1.71 | (1.52,1.93) | 5.32 | (4.75,5.95) | 0.0013 | (0.0009,0.0017) |
| 1940-1944 | 1.56 | (1.39,1.75) | 4.71 | (4.23,5.23) | 0.0023 | (0.0017,0.0031) |
| 1945-1949 | 1.40 | (1.25,1.56) | 4.13 | (3.73,4.57) | 0.0039 | (0.0029,0.0052) |
| 1950-1954 | 1.19 | (1.08,1.32) | 3.42 | (3.11,3.76) | 0.0061 | (0.0046,0.0082) |
| 1955-1959 | 1.14 | (1.03,1.25) | 3.10 | (2.84,3.39) | 0.0103 | (0.0077,0.0139) |
| 1960-1964 | 1.08 | (0.98,1.18) | 2.72 | (2.50,2.97) | 0.0176 | (0.0132,0.0236) |
| 1965-1969 | 1.02 | (0.93,1.12) | 2.35 | (2.17,2.55) | 0.0305 | (0.0228,0.0407) |
| 1970-1974 | 0.96 | (0.88,1.04) | 1.99 | (1.84,2.15) | 0.0518 | (0.0389,0.0690) |
| 1975-1979 | 0.92 | (0.85,1.00) | 1.72 | (1.60,1.85) | 0.0851 | (0.0640,0.1131) |
| 1980-1984 | 0.93 | (0.86,1.01) | 1.59 | (1.48,1.70) | 0.1430 | (0.1080,0.1894) |
| 1985-1989 | 0.94 | (0.88,1.01) | 1.48 | (1.39,1.57) | 0.2333 | (0.1771,0.3073) |
| 1990-1994 | 0.89 | (0.84,0.95) | 1.25 | (1.19,1.32) | 0.3673 | (0.2814,0.4795) |
| 1995-1999 | 0.93 | (0.88,0.98) | 1.12 | (1.07,1.17) | 0.5963 | (0.4606,0.7720) |
| 2000-2004 | 1.00 | | 1.00 | | 1.00 | |
| 2005-2009 | 1.00 | (0.95,1.05) | 0.87 | (0.83,0.91) | 1.4340 | (0.7960,2.5831) |
| 2010-2014 | 0.93 | (0.89,0.99) | 0.73 | (0.69,0.77) | 2.3850 | (0.7603,7.4820) |
| 2015-2019 | 0.81 | (0.76,0.87) | 0.64 | (0.59,0.68) | 3.0559 | (0.4083,22.8689) |

**Supplementary Table 3.** Sensitivity analysis of estimated net drifts, age-specific local drifts, and period rate ratios for analgesic prescribing using the ≥1-prescription definition.

|  | NSAIDs  Est. (95% CI) | | Paracetamol  Est. (95% CI) | | Opioids  Est. (95% CI) | |
| --- | --- | --- | --- | --- | --- | --- |
| Net Drift (% per year) | | | | | | |
|  | -0.01 | (-0.09,0.06) | -0.71 | (-0.80,-0.63) | 6.76 | (6.49,7.03) |
| Age-specific trends (% per year) | | | | | | |
| 01-05 | -0.07 | (-0.34,0.20) | -0.71 | (-0.98,-0.43) | -4.65 | (-8.03,-1.15) |
| 06-10 | 0.29 | (0.06,0.52) | -0.82 | (-1.05,-0.60) | 0.21 | (-2.11,2.60) |
| 11-15 | 0.68 | (0.46,0.90) | -0.60 | (-0.82,-0.38) | 3.27 | (1.64,4.94) |
| 16-20 | 0.56 | (0.35,0.77) | -0.64 | (-0.84,-0.43) | 5.70 | (4.76,6.64) |
| 21-25 | 0.36 | (0.16,0.56) | -0.54 | (-0.74,-0.34) | 6.17 | (5.46,6.89) |
| 26-30 | 0.44 | (0.24,0.64) | -0.28 | (-0.47,-0.08) | 6.53 | (5.92,7.15) |
| 31-35 | 0.49 | (0.29,0.68) | -0.22 | (-0.41,-0.02) | 6.88 | (6.34,7.42) |
| 36-40 | 0.13 | (-0.05,0.32) | -0.70 | (-0.89,-0.51) | 7.10 | (6.62,7.58) |
| 41-45 | -0.08 | (-0.27,0.11) | -1.00 | (-1.20,-0.80) | 7.38 | (6.92,7.83) |
| 46-50 | -0.11 | (-0.31,0.08) | -0.97 | (-1.18,-0.77) | 7.55 | (7.13,7.98) |
| 51-55 | -0.06 | (-0.26,0.15) | -0.74 | (-0.95,-0.53) | 7.57 | (7.16,7.99) |
| 56-60 | -0.46 | (-0.69,-0.23) | -1.00 | (-1.24,-0.77) | 7.19 | (6.77,7.61) |
| 61-65 | -0.84 | (-1.09,-0.59) | -1.25 | (-1.50,-1.00) | 7.24 | (6.82,7.67) |
| 66-70 | -1.08 | (-1.36,-0.80) | -1.43 | (-1.70,-1.15) | 7.78 | (7.35,8.22) |
| 71-75 | -0.64 | (-0.95,-0.32) | -0.75 | (-1.06,-0.44) | 9.10 | (8.64,9.56) |
| 76-80 | -0.01 | (-0.39,0.35) | 0.15 | (-0.21,0.52) | 10.88 | (10.35,11.41) |
| 81-85 | 0.64 | (0.10,1.19) | 1.12 | (0.59,1.65) | 13.09 | (12.12,14.06) |
| Period Rate Ratio (RR) | | | | | | |
| 2001-2005 | 1.00 (ref.) | | 1.00 (ref.) | | 1.00 (ref.) | |
| 2006-2010 | 0.97 | (0.96,0.99) | 0.95 | (0.94,0.96) | 1.98 | (1.91,2.05) |
| 2011-2015 | 1.01 | (1.00,1.02) | 0.96 | (0.95,0.97) | 2.52 | (2.43,2.62) |
| 2016-2020 | 0.98 | (0.97,0.99) | 0.88 | (0.87,0.89) | 2.74 | (2.63,2.85) |

**Supplementary Figure 1.** Age-period-cohort patterns of analgesic utilization in Taiwan (2001–2020).

(a)	(b)	(c)

(d)	(e)	(f)

(g)	(h)	(i)

The panels display the prevalence of NSAIDs (a–c), Paracetamol (d–f), and Opioids (g–i). The left column (a, d, g) illustrates age-specific prevalence rates stratified by calendar period. The middle column (b, e, h) shows temporal trends across calendar periods by age group. The right column (c, f, i) depicts utilization patterns by birth cohort.
